# Cocaine reward and locomotion stimulation in mice with reduced dopamine transporter expression

**DOI:** 10.1186/1471-2202-8-42

**Published:** 2007-06-21

**Authors:** Michael R Tilley, Barbara Cagniard, Xiaoxi Zhuang, Dawn D Han, Narry Tiao, Howard H Gu

**Affiliations:** 1 Department of Pharmacology The Ohio State University College of Medicine, Columbus, OH, USA; 2 Department of Neurobiology, Pharmacology and Physiology, University of Chicago, Chicago, IL, USA; 3 Department of Psychiatry, The Ohio State University College of Medicine, 333 West 10th Avenue, Columbus, OH, USA

## Abstract

**Background:**

The dopamine transporter (DAT) plays a critical role in regulating dopamine neurotransmission. Variations in DAT or changes in basal dopaminergic tone have been shown to alter behavior and drug responses. DAT is one of the three known high affinity targets for cocaine, a powerful psychostimulant that produces reward and stimulates locomotor activity in humans and animals. We have shown that cocaine no longer produces reward in knock-in mice with a cocaine insensitive mutant DAT (DAT-CI), suggesting that cocaine inhibition of DAT is critical for its rewarding effect. However, in DAT-CI mice, the mutant DAT has significantly reduced uptake activity resulting in elevated basal dopaminergic tone, which might cause adaptive changes that alter responses to cocaine. Therefore, the objective of this study is to determine how elevated dopaminergic tone affects how mice respond to cocaine.

**Results:**

We examined the cocaine induced behavior of DAT knockdown mice that have DAT expression reduced by 90% when compared to the wild type mice. Despite a dramatic reduction of DAT expression and marked elevation in basal dopamine tone, cocaine produced reward, as measured by conditioned place preference, and stimulated locomotor activity in these mice.

**Conclusion:**

A reduction in DAT expression and elevation of dopaminergic tone do not lead to adaptive changes that abolish the rewarding and stimulating effects of cocaine. Therefore, the lack of reward to cocaine observed in DAT-CI mice is unlikely to have resulted from the reduced DAT activity but instead is likely due to the inability of cocaine to block the mutated DAT and increase extracellular dopamine. This study supports the conclusion that the blockade of DAT is required for cocaine reward and locomotor stimulation.

## Background

Cocaine is a powerful psychostimulant and an addictive drug of abuse. Considerable evidence indicates that the blockade of the dopamine transporter (DAT) by cocaine and the subsequent elevation of extracellular dopamine (DA) primarily mediate the stimulating and rewarding effects of cocaine [1-4]. DA is an important neurotransmitter involved in many critical functions including motor control, emotion, motivation, memory, and reward [5]. The DA transporter is responsible for DA reuptake and recycling, and thus plays a critical role in maintaining DA homeostasis and in regulating DA neurotransmission. Variations or polymorphisms in the DAT gene have been associated with behavioral changes and modified drug response. For instance, it has been shown that the 9-repeat allele of a 3' variable number tandem repeat polymorphism in the DAT gene is associated with lower basal DA tone and greater DA release in response to nicotine [6]. More significantly, the 9-repeat allele has also been associated with attention deficit/hyperactivity disorder and increased aggression [7,8].

There is also evidence that changes to DAT in animals can affect their drug responses and behavior. It is widely believed that the addictive psychostimulants elevate locomotor activity and produce reward by activating the dopaminergic system. However, cocaine still produces reward in dopamine transporter knockout mice (DAT-KO mice) [9-11]. Importantly, DAT-KO mice show additional differences from wild type mice. The selective serotonin transporter (SERT) inhibitor fluoxetine and the selective norepinephrine transporter (NET) inhibitor nisoxetine do not produce reward in wild type mice, but both drugs produce reward in DAT-KO mice [12-14]. Fluoxetine and reboxetine (another NET selective inhibitor) have also been reported to increase extracellular DA levels in the nucleus accumbens in DAT-KO mice, but not in wild type mice [13,15,16]. It is likely that the absence of DAT in DAT-KO mice has altered neuronal signaling pathways. Therefore, it is not clear how the modulation of DAT would affect cocaine induced reward and locomotor activity.

To avoid the adaptive changes seen in DAT-KO mice, we previously generated a knock-in mouse line with a cocaine-insensitive DAT (DAT-CI) [17]. In DAT-CI mice, cocaine decreases locomotor activity and does not produce reward as measured by conditioned place preference (CPP) [17]. Our results suggest that the blockade of DAT is required for cocaine induced reward and locomotor stimulation. In DAT-CI mice however, the modified DAT has reduced transport function resulting in elevated extracellular DA levels and hyperactivity. Therefore, it is a concern that cocaine's inability to produce reward or to increase locomotor activity might be due to increased DA basal tone or other adaptations caused by the reduced DA transport function in DAT-CI mice.

A DAT knock-down mouse line (DAT-KD) has been generated which has a 90% reduction in DAT expression compared to the wild type mice [18]. DAT-KD mice display significantly altered behaviors, such as hyperactivity and slower habituation to a novel environment. Importantly, amphetamine, which increases locomotion in wild type mice, reduces locomotor activity in DAT-KD mice [18]. DAT-KD mice also exhibit differences from wild type mice in their response to sweet rewards [19,20]. These studies indicate that alterations in DAT expression can change an animal's behavior and modify its response to drugs including psychostimulants. DAT-KD mice provide a good animal model to test how reduced DAT expression affects cocaine-induced animal behaviors.

In the current study, we compared the cocaine response in DAT-KD mice and wild type mice with two behavioral tests: drug induced locomotion and CPP. Our results revealed the effects of reduced DAT expression on cocaine-elicited animal behaviors.

## Results

### Locomotion stimulation

**Figure 1:**
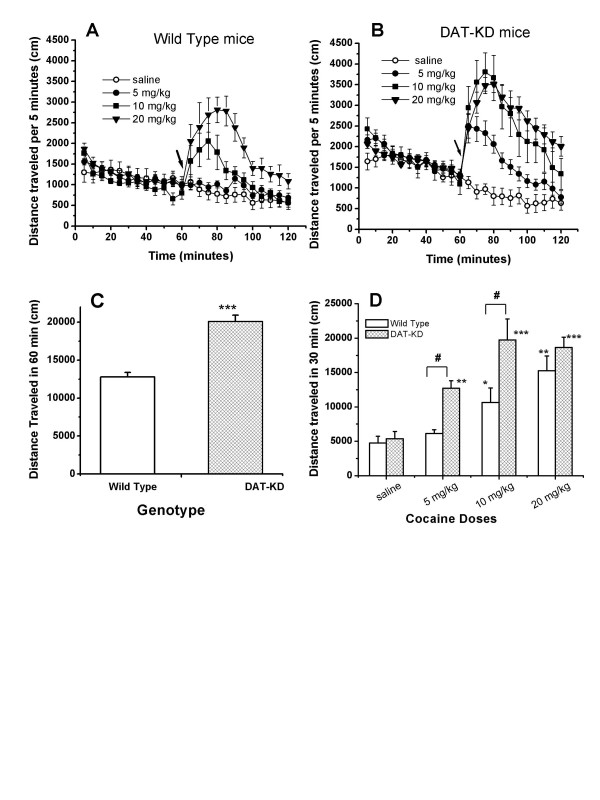
**Effect of cocaine on locomotor activity in DAT-KD mutant mice compared to WT mice**. Mice were habituated to the locomotor test chamber for 60 minutes. Cocaine or saline were injected (ip) and mice were returned to the test chamber and monitored for another 60 min. A) and B): Time course of locomotor activity of WT mice and DAT-KD mice. Saline, 5 mg/kg, 10 mg/kg or 20 mg/kg cocaine was given at the time indicated by the arrows. Data shown are average distance traveled in 5 min. C) Total distance traveled in 60 minutes for wild type and DAT-KD mice during their habituation to the chambers before drug or saline injection. DAT-KD mice are significantly more active than wild type ice (***, p < 0.001, t-test). D) Total distance traveled in 30 min after the injection of saline or 5, 10, or 20 mg/kg cocaine with 6 – 8 mice in each group. Two-way ANOVA was performed. Cocaine significantly increased locomotor activities in both genotypes of mice (for drug effect, F_3,66 _= 22.38, p < 0.001) and had greater effect on DAT-KD mice than on the WT mice (for genotype, F_1,66 _= 19.57, p < 0.001). Error bars represent standard error of means. Post hoc Bonferroni tests versus saline: *, p < 0.05; **, p < 0.01; ***, p < 0.001. Comparing between the two genotypes, 5 mg/kg and 10 mg/kg cocaine had a greater effect on locomotor activity in the DAT-KD mice than in wild type mice (#, p < 0.01).

First, we examined the effects of cocaine or saline injection on mouse locomotor activity. WT and DAT-KD mice were placed in an open field box (25 cm × 25 cm) and locomotor activity was recorded for 60 minutes. After the habituation period, cocaine or saline were injected and mouse locomotor activities were monitored for another 60 minutes. Figure 1A and 1B show the time course of the response of DAT-KD and wild type mice, respectively, to saline, 5 mg/kg, 10 mg/kg and 20 mg/kg cocaine. DAT-KD mice displayed higher basal locomotor activity (p < 0.001, t-test) than their wild type littermates (Fig. 1C). This observation is consistent with previously published results [18]. Fig. 1D shows the total distance traveled in 30 min after injection of saline or different doses of cocaine. Two way ANOVA detected a main effect of drug (F_3,66 _= 22.38, p < 0.001) and significant difference between genotypes (F_1,66 _= 19.57, p < 0.001); but the drug-genotype interaction did not reach a significant level (F_3,66 _= 2.686, p = 0.053). The data were further analyzed with post hoc Bonferroni tests. Comparing the two genotypes of mice, we found no difference between WT and DAT-KD mice in distance traveled after saline injection (p > 0.05), despite the higher basal locomotor activity in DAT-KD mice. However, 5 mg/kg and 10 mg/kg cocaine had a greater stimulating effect on locomotor activity in the DAT-KD mice (p < 0.01 and p < 0.001), while there was no significant difference in the effects of 20 mg/kg cocaine on the two genotypes of mice (p > 0.05). For the cocaine effect, post hoc tests indicated that 5, 10, and 20 mg/kg cocaine significantly stimulated locomotor activity compared to saline in the DAT-KD mice (p < 0.01, p < 0.001, and p < 0.001). In the wild type mice, 5 mg/kg cocaine did not significantly stimulate locomotor activity, while 10 and 20 mg/kg cocaine did (p > 0.05, p < 0.001, and p < 0.001 respectively).

### Conditioned place preference

**Figure 2:**
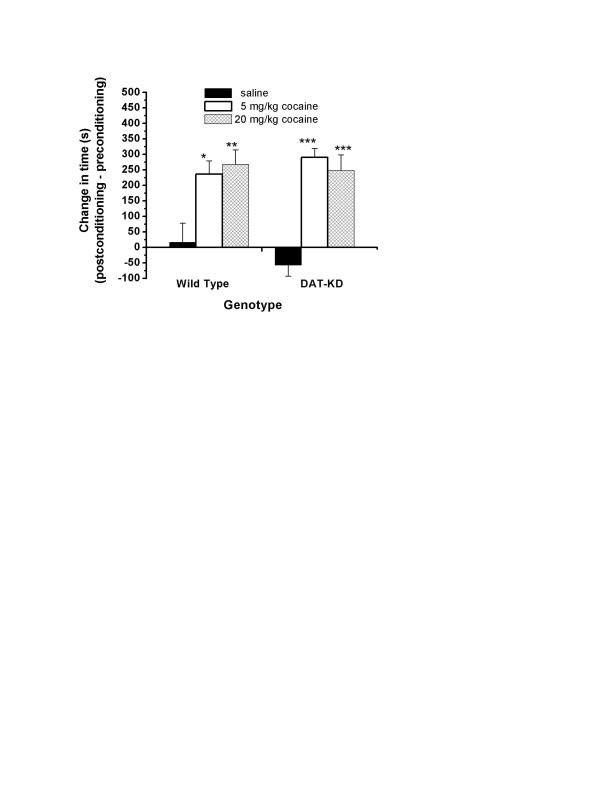
**Cocaine induced CPP in DAT-KD mutant mice and WT mice**. CPP is represented by the difference in the time mice spend in the drug-paired chamber between pre-conditioning day and post-conditioning day. Both 5 and 20 mg/kg cocaine produced significant CPP in both WT mice and DAT-KD mice (for drug effect, F_2,1 _= 24.25, p < 0.0001). Eight mice were examined in each group. Error bars represent standard error of means. Post hoc Bonferroni tests versus saline: *, p < 0.05; **, p < 0.01; ***, p < 0.001.

Next, we tested whether cocaine can still produce reward in DAT-KD mice using the CPP test. As shown in Fig. 2, 5 mg/kg and 20 mg/kg cocaine significantly increased the time spent in cocaine-paired chamber by both wild type and DAT-KD mice. Statistical analysis with ANOVA indicated a main drug effect (F_2,40 _= 24.25, p < 0.0001), the lack of a difference between genotypes (F_1,40 _= 0.1180, p > 0.05) and the lack of a drug-genotype interaction (F_2,40 _= 0.8827, p > 0.05). Post-hoc Bonferroni analysis showed the increase in time spent in the cocaine-paired chamber was significant at 5 mg/kg and 20 mg/kg cocaine for both wild type (p < 0.05 and 0.01 respectively) and DAT-KD mice (p < 0.001 and 0.001 respectively). The time mice spent in the unpaired chamber decreased in the cocaine treated groups (data not shown). Therefore, the rewarding effect of cocaine is preserved in DAT-KD mice in which the activity of DAT, the primary target of cocaine, is dramatically reduced.

## Discussion

The dopamine transporter is a key component in the regulation of DA neurotransmission. Most evidence suggests that the blockade of DAT and the subsequent increase in extracellular DA primarily mediate the stimulating and rewarding effects of cocaine. However, the persistence of the rewarding effect of cocaine in DAT-KO mice suggests a mechanism of DAT-independent cocaine reward, which might be due to the extensive adaptive changes in DAT-KO mice. To test whether the DAT-independent cocaine reward also plays a role in normal mice, we made a knock-in mouse line with the endogenous DAT replaced by a cocaine-resistant DAT mutant. In these DAT-CI mice, cocaine suppresses locomotion and does not produce reward, supporting the hypothesis that cocaine blockade of DAT is required for cocaine reward in normal mice [17]. However, DAT activity in DAT-CI mice is significantly lower than that in wild type mice, and changes in DAT have been shown to alter behaviors or drug responses. For example, reduced DAT activity in DAT-KD mice has been shown to alter behavioral responses to amphetamine, another psychostimulant that impacts the dopaminergic system [18]. It remains a concern that in DAT-CI mice the lack of cocaine response might be due to compensatory changes from the lowered DAT activity. Therefore, we studied cocaine effects on DAT-KD mice.

The DAT-KO and DAT-CI mice were generated using ES cells from 129Sv/J mice and crossed with C57BL6 mice, thus they have a mixed genetic background [17]. Several studies used mice that were backcrossed with C57BL6 mice for many generations [17]. The original DAT-KD mice were generated in 129Sv/J background but this strain does not respond well in many tests that are used to evaluate psychostimulant effects. Therefore, the DAT-KD mice used in this study were backcrossed to C57BL/6J for 8 to 9 generations and thus their background matches with the backgrounds of both the DAT-CI mice and DAT-KO mice. This is important because it has been shown that cocaine's effects are not the same in different strains of mice [21,22]. In addition, DAT-KO mice backcrossed to different strain backgrounds also respond to cocaine differently [23].

In DAT-KD mice, DAT activity is reduced to 10% of the wild type level, resulting in a hyperdopaminergic tone and increased locomotor activity [18]. In addition, DAT-KD mice have an altered response to amphetamine, which might be due to an altered balance between DA autoreceptor and heteroreceptor functions [18]. Amphetamine elevates extracellular DA by interacting with DAT and, more importantly, with the vesicular monoamine transporter, inhibiting DA uptake, and inducing DA release from DA containing vesicles to the cytosol through the vesicular monoamine transporter and to the extracellular space through DAT [5]. In contrast, cocaine elevates extracellular DA by simply binding to DAT and inhibiting DA reuptake. Despite the vastly different mechanisms of action, amphetamine and cocaine both increase DA in the synapse and enhance dopaminergic neurotransmission, thereby stimulating locomotor activity and producing reward in animals. The fact that amphetamine produces locomotor inhibition instead of stimulation in DAT-KD mice suggested the possibility that cocaine effects might also be altered in these mice.

Fig. 1 shows that DAT-KD mice had considerably higher baseline locomotor activity than WT mice before drug or saline injection and this difference was statistically significant (Fig. 1C). This result confirms our previous observation [18] and is consistent with the hyperdopaminergic tone in these mice [18]. Fig. 1 also shows that cocaine clearly increased locomotor activity in DAT-KD mice as well as in WT mice. While cocaine was capable of increasing locomotor activity in both genotypes, there were differences in each genotype's response. For instance, both 5 and 10 mg/kg cocaine produced stronger locomotor stimulation in DAT-KD mice than it did in wild type mice (Fig. 1D). The reason for this difference is not clear but could be due to presynaptic or postsynaptic adaptive changes, such as reduced D2 autoreceptor signaling, in response to the hyperdopaminergic tone in DAT-KD mice. It is also not clear why the amphetamine effect is altered in DAT-KD mice while the cocaine effect is maintained. Our results demonstrate that a dramatically reduced DAT activity does not lead to a loss or a reduction of the stimulating effect of cocaine. This is in contrast to observations of DAT-KO mice and DAT-CI mice. In heterozygous DAT-KO mice, DAT activity is reduced by 50% and cocaine stimulates locomotion as well as in WT mice; while in homozygous DAT-KO mice, DAT is absent and cocaine has no effect on locomotion [24]. In DAT-CI mice, the mutated DAT is over 50-fold less sensitive to cocaine inhibition but has lower uptake activity. Instead of stimulation, cocaine suppresses locomotor activity in DAT-CI mice [17]. The fact that cocaine retains these effects in both heterozygous DAT-KO mice and DAT-KD mice indicates that the reduction of DAT expression and thus DAT activity to 50% and 10% of wild type mice does not by itself lead to adaptive changes that abolish the stimulating effect of cocaine and in fact, 10% DAT activity seems to be sufficient for the preservation of many normal functions, such as some cocaine responses. Therefore, the locomotor suppression observed in DAT-CI mice is not due to reduced DA clearance or changes in DA homeostasis but is due to the lack of cocaine inhibition of DAT and to cocaine effects on other cocaine targets. Cocaine inhibition of DAT, SERT, and NET and the resulting enhancement of neurotransmission in all three systems may contribute to the locomotor effect in wild type mice. Cocaine inhibition of SERT and/or NET in the absence of DAT inhibition in DAT-CI mice leads to locomotor suppression. It is not clear whether cocaine inhibition of SERT or NET enhances or dampens locomotor stimulation in wild type mice where DAT is also inhibited. Further studies are being performed to assess the role of SERT and NET in locomotor suppression.

Cocaine and other addictive drugs are known to produce reward in humans as well as in mice and other animals. CPP tests are commonly used to measure the rewarding properties of drugs. In this behavioral test, drug administration to animals is repeatedly paired with a set of environmental cues. When allowed to explore freely, the animals spend more time in the environment paired with a drug that produces reward.

Previously we have shown that up to 20 mg/kg cocaine is not enough to inhibit the cocaine-insensitive mutant DAT and elevate extracellular DA in DAT-CI mice [17]. We have also shown that cocaine lost its ability to produce reward in DAT-CI mice while amphetamine still produces CPP in these mice. Therefore, we conclude that the reward pathway in DAT-CI mice is functional and the lack of cocaine-induced reward in DAT-CI mice is due to the inability of cocaine to block the modified DAT. However, DAT activity in DAT-CI mice is significantly lower than that in WT mice. The DA clearance rate in DAT-CI mice is between those of DAT-KD mice and heterozygous DAT-KO mice [17]. It remains a concern that lowered DAT activity might lead to neuroadaptations that may alter cocaine effects. Thus, we examined whether cocaine is still able to produce reward in DAT-KD mice that have only 10% of wild type DAT expression. Fig. 2 shows that cocaine still produced robust CPP in DAT-KD mice. In addition, cocaine produces reward in heterozygous DAT-KO mice [9-11]. These results demonstrate that the reduction of DAT activity and elevated dopaminergic tone does not abolish cocaine's rewarding effect in mice.

Several mouse models have been generated with alterations to the DAT gene, providing excellent tools to study the functions of DAT and the mechanism of cocaine effects: DAT-KD mice [18] and heterozygous DAT-KO mice [24] with DAT expression reduced to 10% and 50% of the wild type level respectively, DAT-CI mice [17] with a cocaine-insensitive DAT mutant that is functional but with reduced uptake activity, and homozygous DAT-KO mice [24] with DAT completely deleted. Heterozygous DAT-KO mice [24]and DAT-KD mice have significantly elevated DA tone but in these mice DAT is blocked by cocaine and the mice respond to cocaine similarly to wild type mice. The adaptive changes to the elevated dopaminergic tone in these mice are likely to be moderate. The changes do not abolish cocaine's ability to produce reward or to stimulate locomotor activity. Therefore, it is likely that the mechanism of cocaine action in these mice is similar to that in wild type mice.

In DAT-CI mice, DA uptake activity is reduced and the basal DA tone is also significantly elevated [17]. Since the extent of basal DA elevation in DAT-CI mice is between those in heterozygous DAT-KO and DAT-KD mice [17], it is reasonable to assume that the adaptive changes in DAT-CI mice are also moderate and do not interrupt the drug reward pathway. We have shown that amphetamine still produces reward in DAT-CI mice suggesting a functional reward pathway [17]. Although the elevated basal DA tone is due to reduced DAT activity in all mouse lines, the causes of the reduction are different; reduced expression of wild type DAT, in heterozygous DAT-KO and DAT-KD mice, versus normal expression of a mutant DAT with lower uptake activity in DAT-CI mice. Therefore, it is still possible that the mutated DAT in DAT-CI mice may cause unique adaptive changes, by an unknown mechanism, which could abolish cocaine reward and locomotor stimulation.

In contrast, in homozygous DAT-KO mice DAT is completely lacking. Intriguingly, the elimination of DAT, the consequent severe alteration of DA homeostasis and related pathways, and the tremendous adaptive changes still fail to abolish the rewarding effect of cocaine [24]. However, the mechanism of cocaine reward in DAT-KO mice is very different from that in wild type mice. For example, inhibition of SERT or NET with selective inhibitors elevates DA levels in the nucleus accumbens and produces reward in DAT-KO mice but not in wild type mice [24]. These results indicate that the complete deletion of DAT significantly alters the mechanism of cocaine effects. It seems that cocaine-induced DA elevation in the nucleus accumbens is still critical and the signaling from serotoninergic or noradrenergic systems to the dopaminergic system might be altered. The exact mechanism is not clear and currently under intensive investigation.

## Conclusion

In summary, genetically modified mouse strains with a reduction in DAT function by 50% (heterozygous DAT-KO mice) and 90% (DAT-KD mice) retain the stimulating and rewarding effects of cocaine. These results are consistent with the idea that reduced DAT activity in DAT-CI mice does not lead to adaptive changes that abolish the stimulating and rewarding effects of cocaine. Therefore, the lack of cocaine reward and locomotor stimulation in DAT-CI mice is unlikely due to reduced DAT activity or the resulting hyperdopaminergic tone, but due to the inability of cocaine to inhibit the modified DAT. This investigation lends further support to the conclusion that cocaine inhibition of DAT is necessary for the rewarding and stimulating effects of cocaine.

## Methods

### Animals

The DAT-KD mice were generated by the insertion of a DNA construct containing the neomycin-resistant gene and other elements into the promoter region of DAT gene in an attempt to control DAT expression [18]. This promoter modification results in a 90% reduction in DAT expression [18]. Mice used in this study were produced from the breeders that have been backcrossed with C57BL/6J mice for 8–9 generations. Heterozygous male and female DAT-KD mice were bred to produce homozygous DAT-KD mice and their WT littermates used in the experiments. The genotypes of the mice used in these experiments were determined by PCR using forward primer TGGGGTCCACATACAAATGATGA and reverse primer ACACGTGGCAGATTCATAGGTA. Only male mice between 10 and 14 weeks of age were used in this study. Mice were group housed, with 3 – 5 mice per cage, on a 12 hour day/night cycle with lights off at 6 pm. Food and water was provided *ad libitum*. All procedures were conducted in accordance with the animal use committee at The Ohio State University. Experiments were performed between 9:00 am and 5:00 pm. Only drug naïve animals were used in experiments and separate animals were used for the locomotor and CPP studies.

### Drugs

The drug supply program of National Institute on Drug Abuse, NIH (Bethesda, MD), kindly supplied cocaine HCL. Doses of 5 mg/kg, 10 mg/kg and 20 mg/kg were given for the locomotion studies and doses of 5 mg/kg and 20 mg/kg were used for the CPP studies. Vehicle (0.9% NaCl) and cocaine were administered through intraperitoneal (i.p.) injection.

### Locomotion tests

Locomotion tests were performed in acrylic test chambers measuring 25 cm(W) × 25 cm(L) × 25 cm(H) that are inside a sound-attenuating behavioral test enclosure. Mice were placed in the chamber without drugs for 60 min. The total distance traveled during this time was used to determine the basal activity. Then cocaine or vehicle was administered and mouse locomotor activities were monitored for another 60 minutes. Cumulative distance traveled and speed was recorded by a video-based monitoring system, Limelight (Actimetrics, Evanston, IL). The stimulatory effects of cocaine peaked between 10 and 20 minutes, thus we used distance traveled within the first 30 minutes after injection as a measurement of cocaine effect on locomotion.

### Conditioned place preference

CPP was performed as previously described [17]. In this setup, we used a three-chambered box with the two end chambers differing in wall patterns and floor materials. One chamber had a thin wavy pattern on a white background on the walls and thin-mesh flooring, while the other chamber had a thick grid pattern on a white background on the walls and thick-mesh flooring. The middle chamber, used as a neutral initial placement point, had gray walls and a slick, acrylic floor. On the pre-conditioning day (day 1) mice were locked in the middle chamber for 30 seconds, then allowed to freely explore the entire apparatus for 30 min. Movement and time spent in each chamber was recorded with the video-based monitoring system LimeLight. Mice did not show a significant preference for either end chamber. Mice were randomly paired to one end chamber and randomly divided into saline control and cocaine-injected groups. On days 2, 4, 6, and 8, the mice were injected with saline and confined in one of the two end chambers for 30 min; and on days 3, 5, 7, and 9, the mice were injected with cocaine or saline and confined to the opposite end chamber for 30 minutes. On the test day (day 10), the mice were confined to the central chamber for 30 seconds, and then allowed free access to both end chambers for 30 min. The time spent in each of the end chambers was recorded. The difference between the time spent in the paired chamber on pre-conditioning day and time spent in the same chamber on post-conditioning day was calculated and presented.

### Data analysis

The experimental data were analyzed by two-way ANOVA and post hoc Bonferroni tests using GraphPad Prism (GraphPad Software, Inc. San Diego, CA)

## Authors' contributions

MT carried out the behavioral tests, analyzed data, designed experiments and participated in drafting the manuscript. BC and XZ generated and backcrossed the DAT-KD mice. DH and NT continued the backcrossing of DAT-KD mice, performed genotyping, and maintained the mouse colony. HG conceived of the study, analyzed data, and was responsible for its design and coordination, and for drafting the manuscript. All authors read and approved the final manuscript.

## References

[B1] Wise RA, Bozarth MA (1987). A psychomotor stimulant theory of addiction. Psychol Rev.

[B2] Di Chiara G, Imperato A (1988). Drugs abused by humans preferentially increase synaptic dopamine concentrations in the mesolimbic system of freely moving rats. Proceedings of the National Academy of Sciences of the United States of America.

[B3] Bergman J, Madras BK, Johnson SE, Spealman RD (1989). Effects of cocaine and related drugs in nonhuman primates. III. Self-administration by squirrel monkeys. J Pharmacol Exp Ther.

[B4] Kuhar MJ, Ritz MC, Boja JW (1991). The dopamine hypothesis of the reinforcing properties of cocaine. Trends in Neurosciences.

[B5] Nestler EJ, Hyman SE, Malenka RC (2001). Molecular neuropharmacology : a foundation for clinical neuroscience.

[B6] Brody AL, Mandelkern MA, Olmstead RE, Scheibal D, Hahn E, Shiraga S, Zamora-Paja E, Farahi J, Saxena S, London ED, McCracken JT (2006). Gene variants of brain dopamine pathways and smoking-induced dopamine release in the ventral caudate/nucleus accumbens. Arch Gen Psychiatry.

[B7] Todd RD, Huang H, Smalley SL, Nelson SF, Willcutt EG, Pennington BF, Smith SD, Faraone SV, Neuman RJ (2005). Collaborative analysis of DRD4 and DAT genotypes in population-defined ADHD subtypes. J Child Psychol Psychiatry.

[B8] Gerra G, Garofano L, Pellegrini C, Bosari S, Zaimovic A, Moi G, Avanzini P, Talarico E, Gardini F, Donnini C (2005). Allelic association of a dopamine transporter gene polymorphism with antisocial behaviour in heroin-dependent patients. Addict Biol.

[B9] Rocha BA, Fumagalli F, Gainetdinov RR, Jones SR, Ator R, Giros B, Miller GW, Caron MG (1998). Cocaine self-administration in dopamine-transporter knockout mice [see comments] [published erratum appears in Nat Neurosci 1998 Aug;1(4):330]. Nat Neurosci.

[B10] Sora I, Wichems C, Takahashi N, Li XF, Zeng Z, Revay R, Lesch KP, Murphy DL, Uhl GR (1998). Cocaine reward models: conditioned place preference can be established in dopamine- and in serotonin-transporter knockout mice. Proc Natl Acad Sci U S A.

[B11] Medvedev IO, Gainetdinov RR, Sotnikova TD, Bohn LM, Caron MG, Dykstra LA (2005). Characterization of conditioned place preference to cocaine in congenic dopamine transporter knockout female mice. Psychopharmacology (Berl).

[B12] Hall FS, Li XF, Sora I, Xu F, Caron M, Lesch KP, Murphy DL, Uhl GR (2002). Cocaine mechanisms: enhanced cocaine, fluoxetine and nisoxetine place preferences following monoamine transporter deletions. Neuroscience.

[B13] Mateo Y, Budygin EA, John CE, Jones SR (2004). Role of serotonin in cocaine effects in mice with reduced dopamine transporter function. Proc Natl Acad Sci U S A.

[B14] Budygin EA, Brodie MS, Sotnikova TD, Mateo Y, John CE, Cyr M, Gainetdinov RR, Jones SR (2004). Dissociation of rewarding and dopamine transporter-mediated properties of amphetamine. Proc Natl Acad Sci U S A.

[B15] Carboni E, Spielewoy C, Vacca C, Nosten-Bertrand M, Giros B, Di Chiara G (2001). Cocaine and amphetamine increase extracellular dopamine in the nucleus accumbens of mice lacking the dopamine transporter gene. J Neurosci.

[B16] Shen HW, Hagino Y, Kobayashi H, Shinohara-Tanaka K, Ikeda K, Yamamoto H, Yamamoto T, Lesch KP, Murphy DL, Hall FS, Uhl GR, Sora I (2004). Regional Differences in Extracellular Dopamine and Serotonin Assessed by In Vivo Microdialysis in Mice Lacking Dopamine and/or Serotonin Transporters. Neuropsychopharmacology.

[B17] Chen R, Tilley MR, Wei H, Zhou F, Zhou FM, Ching S, Quan N, Stephens RL, Hill ER, Nottoli T, Han DD, Gu HH (2006). Abolished cocaine reward in mice with a cocaine-insensitive dopamine transporter. Proc Natl Acad Sci U S A.

[B18] Zhuang X, Oosting RS, Jones SR, Gainetdinov RR, Miller GW, Caron MG, Hen R (2001). Hyperactivity and impaired response habituation in hyperdopaminergic mice. Proc Natl Acad Sci U S A.

[B19] Pecina S, Cagniard B, Berridge KC, Aldridge JW, Zhuang X (2003). Hyperdopaminergic mutant mice have higher "wanting" but not "liking" for sweet rewards. J Neurosci.

[B20] Cagniard B, Balsam PD, Brunner D, Zhuang X (2006). Mice with chronically elevated dopamine exhibit enhanced motivation, but not learning, for a food reward. Neuropsychopharmacology.

[B21] Schlussman SD, Ho A, Zhou Y, Curtis AE, Kreek MJ (1998). Effects of "binge" pattern cocaine on stereotypy and locomotor activity in C57BL/6J and 129/J mice. Pharmacol Biochem Behav.

[B22] He M, Shippenberg TS (2000). Strain differences in basal and cocaine-evoked dopamine dynamics in mouse striatum. J Pharmacol Exp Ther.

[B23] Morice E, Denis C, Giros B, Nosten-Bertrand M (2004). Phenotypic expression of the targeted null-mutation in the dopamine transporter gene varies as a function of the genetic background. Eur J Neurosci.

[B24] Giros B, Jaber M, Jones SR, Wightman RM, Caron MG (1996). Hyperlocomotion and indifference to cocaine and amphetamine in mice lacking the dopamine transporter. Nature.

